# Sgttoolbox: Utility for controlling SimpleGazeTracker from Psychtoolbox

**DOI:** 10.3758/s13428-016-0791-4

**Published:** 2016-08-08

**Authors:** Hiroyuki Sogo

**Affiliations:** Faculty of Law and Letters, Ehime University, 3 Bunkyo-cho, Matsuyama, Ehime 790-8577 Japan

**Keywords:** Eye tracking, Open source, Psychtoolbox, Performance test

## Abstract

Eye movement analysis is effective for investigating visual perception and cognition. The cost of conducting eye movement studies has decreased as a result of the recent release of low-cost commercial and open-source eye trackers. However, synchronizing visual stimulus presentation with eye movement recording is still difficult, particularly if the eye tracker does not come with a practical application programming interface. This paper introduces a Matlab/Octave toolbox named Sgttoolbox, which works in conjunction with the widely used experiment control library Psychtoolbox to control a cross-platform open-source eye tracker named SimpleGazeTracker, which is an eye-tracking application of GazeParser software. Hardware and software requirements for Sgttoolbox and its main functions are described. A test of temporal accuracy showed that eye movement sampling frequency was stable when stimulus presentation and recording were performed on a single PC, although better performance was obtained when presentation and recording were performed on separate PCs. Transferring the latest eye position from SimpleGazeTracker to Psychtoolbox script takes 2 to 4 ms on average, which causes a delay in drawing multiple visual stimuli when recording and stimulus presentation were performed on a single PC. When such a transfer delay is not importnat, Sgttoolbox would be a good choice for Psychtoolbox users who wish to conduct eye-tracking studies.

## Introduction: SimpleGazeTracker and Psychtoolbox

Human visual functions such as color sensitivity and spatio-temporal resolution change across the visual field. During daily visual cognitive tasks, we frequently make rapid eye movements called saccades in order to look at visual targets with the central visual field, the spatial resolution of which is the highest in the visual field. Therefore, eye movement measurement is an important technique in the study of human visual cognition, including domains such as space perception, scene recognition, reading, spoken language processing and various clinical studies (Liversedge, Gilchrist & Everling, 2011; van Gompbel, Fischer, Murray & Hill, 2007).

In eye movement studies, recording devices called "eye trackers" are used for precise and accurate measurement. At present, there are many eye trackers of various measurement principals, functionalities, sizes, and prices. For example, the Eyelink series (SR Research Ltd.), iViewX series (SMI GmbH) and Tobii series (Tobii Technology, Ltd) all exemplify high performance commercial eye trackers. They have precision and accuracy for research use, as well as application programming interfaces (APIs) for using eye trackers for experimental programs. A weakness of these products are the starting cost, particularly when introducing high-end models. Recently, several low-cost commercial eye trackers such as The Eyetribe (The Eye Tribe Aps), GazePoint3 (GazePoint), and EyeX (Tobii Technology, Ltd) were released. These eye trackers are sufficient for various experiments where a high sampling rate is not required. In addition to these commercial eye trackers, open-source low-cost eye trackers are also available (Li, Babcock & Parkhurst, 2006; San Austin, Skovsgaard, Mollenbach, Barret, Tall, Hansen & Hansen 2010; Zielinski, 2007). Usually, these open-source eye trackers require a camera unit to capture eye images. Because recent low-cost commercial eye trackers are as inexpensive as decent camera units, open-source eye trackers may not be the most inexpensive current choice for eye tracking; however, they would still be worthy of consideration depending on the required purpose.

GazeParser is an open-source library for eye tracking and gaze data analysis (Sogo, 2013). It supports multiple camera units (OptiTrack USB 2.0 cameras, PointGray Flea3 UBS 3.0 cameras, CameraLink cameras and cameras supported by OpenCV) so that users can select a camera unit suitable for their requirements. GazeParser captures an image of the participant’s eye and detects the center of the pupil as well as the reflection image of the illumination on the cornea (i.e., the Purkinje image). Calibration has to be performed, such that the center of pupil and the Purkinje image are converted to the gaze position on the stimulus presentation screen. After calibration has been performed, GazeParser can continuously capture camera images to calculate gaze position, and records to a data file in Comma-Separated Value (CSV) format. Calibration and recording can be easily controlled from Python-based experimental control libraries such as VisionEgg (Straw, 2008) and PsychoPy (Peirce, 2007, 2008). Performance tests on GazeParser showed that spatial accuracy of gaze position ranged from 0.7° to 1.2°, depending on the participant. The mean sampling interval error was less than 1 ms. In gap/overlap tasks and antisaccade tasks, the mean latency and amplitude of saccades recorded by GazeParser was in agreement with those recorded by the Eyelink system (Sogo, 2013).

Since GazeParser was released, the developer has frequently received requests to make this eye tracker support Psychtoolbox (Brainard, 1997; Pelli, 1997). Psychtoolbox is a widely used experimental control library that runs on Matlab/Octave. Although GazeParser was not originally developed to be compatible with Psychtoolbox, the eye-tracking program of GazeParser can be controlled from non-Python experiment programs, because the eye-tracking program uses TCP/IP connection to exchange commands and data. Therefore, GazeParser’s eye-tracking program can be controlled from Psychtoolbox scripts by sending appropriate commands through TCP/IP. In order to respond to requests for supporting Psychtoolbox, development of a Matlab/Octave toolbox named “SimpleGazeTracker Toolbox” (Sgttoolbox) has been started. Sgttoolbox is available from Sgttoolbox’s project page (http://sgttoolbox.sourceforge.net/). In accordance with the development of Sgttoolbox, the GazeParser’s eye tracking program is separated from GazeParser and has been renamed “SimpleGazeTracker”. SimpleGazeTracker can be downloaded from GazeParser’s project page (http://gazeparser.sourceforge.net/). For those who want to use SimpleGazeTracker from programming languages other than Python and Matlab, the TCP/IP command reference of SimpleGazeTracker is published on the GazeParser project page.

The present paper describes how to set up and use Sgttoolbox and SimpleGazeTracker. In the next section, required operating system (OS), software, computer and camera units for using Sgttoolbox and SimpleGazeTracker are described. Then, the installation procedure for Sgttoolbox and SimpleGazeTracker is explained briefly and an outline of main Sgttoolbox commands is provided. Usage details for Sgttoolbox and SimpleGazeTracker are provided at the project pages of Sgttoolbox and GazeParser, respectively. Finally, the results of performance tests on SimpleGazeTracker’s sampling frequency and communication delay between SimpleGazeTracker and Sgttoolbox are described.

## Hardware and software requirements

### Supported operating systems and camera units

SimpleGazeTracker is developed on Microsoft Windows 7, Windows 10 and Ubuntu 14.04 LTS. Linux distributions other than Ubuntu will be capable of running SimpleGazeTracker if they support SDL2.0, libusb 1.0, and OpenCV 2.4 or 3.x. For simplicity, these OSs are referred to as “Windows” and “Linux” in the rest of this paper. MacOS X had been supported until SimpleGazeTracker version 0.6.6 (released on 10 December 2013), but is not officially supported now.

Four variants of SimpleGazeTracker are currently distributed, which are built with different image-capturing libraries. Table 1 shows names, supported operating systems, cameras, and brief descriptions of these variants. Among them, the OptiTrack version is recommended if Windows PC is available and a sampling frequency of 100 Hz is enough for this purpose. The OptiTrack V100R2 camera has built-in infrared (IR) LED illumination, so separate IR illumination is unnecessary. It can be connected to a PC via USB2.0. If a higher sampling frequency is necessary, the FlyCapture2 and GPC5300 versions are suitable. The FlyCapture2 version works both on Linux and Windows; however, the sampling frequency of the FlyCapture2 version is not as stable as that of the GPC5300 version. For example, while the camera is set to capture 250 frames per second (FPS), only 200–240 frames may be obtained depending on the processing power of the computer. This issue will be mentioned later in the “Performance evaluation” section of this paper. The GPC5300 version with a Bobcat ICL-B0620 (IMPREX Inc.) camera achieved 500 Hz recording in a previous study (Sogo, 2013). A Camera Link interface board made by Interface cooperation is necessary to use the GPC5300 version. The OpenCV version uses the VidoCapture class of OpenCV to capture images. Many USB web cameras work with the VideoCapture class. On Linux, FireWire (IEEE194) cameras which support libdc1394 also work with the VideoCapture class. The OpenCV version is recommended for Linux users who want to use FireWire cameras. Although a USB web camera can be used from the OpenCV version of SimpleGazeTracker, high performance cannot be expected with such cameras. Most of these cameras can capture images at 30 Hz or less. An IR cut filter is mounted in many models to improve color reproduction, while SimpleGazeTracker uses IR illumination. Some cameras automatically control shutter speed, sensitivity, and so on, which may have unexpected influences on recording.

**Table 1:** Hardware setup

Variant	Supported OS, cameras and description
OpenCV	OS: Linux, Windows Camera: Any cameras supported by OpenCV::VideoCapture Description: cheap web cameras can be used, but high performance cannot be expected with such cameras.
FlyCapture2	OS: Linux, Windows Camera: Flea3 FL3-U3-13Y3M-C USB3.0 camera (PointGray, Inc.) Description: high-frequency sampling is possible on Linux, but sampling frequency is not stable.
OptiTrack	OS: Windows Camera: OptiTrack V100R2, V120Slim (NaturalPoint, Inc.) Description: price is not high relative to their sampling rate (100 or 120Hz). V100R2 has integrated LED illumination.
GPC5300	OS: Windows Camera: CameraLink cameras supported by GPC-5300 (Interface, Co.) Description: high-frequency sampling is possible.

*OS* operating system

Sgttoolbox is developed on Matlab2015a (Mathworks Inc.) for Windows10 and Octave 3.8.1 for Ubuntu 14.04LTS. Any other systems that can run Psychtoolbox-3 will be capable of running Sgttoolbox.

Sgttoolbox and SimpleGazeTracker can be run either on a single PC (Single-PC setup) or on two separate PCs (Dual-PC setup). SimpleGazeTracker and Sgttoolbox use TCP/IP connection to send and receive data. Therefore, a LAN port is necessary to run SimpleGazeTracker and Sgttoolbox in Dual-PC setup. Wireless LAN is not recommended because it is generally slower than wired LAN. High performance CPU and graphics chips are required to run SimpleGazeTracker and Sgttoolbox in Single-PC setup.

### Other requirements

Using IR illumination and pass filer is highly recommended for reliable data recording. Most Sgttoolbox users would plan to record gaze position while observing visual stimuli drawn by Psychtoolbox. Visual stimuli are reflected on an observer’s cornea, which may disturb pupil detection from captured camera images. Inserting IR pass filter in front of the camera sensor and illuminating an observer’s eye using IR light reduce undesirable corneal reflection of visual stimuli. OptiTrack cameras (V100R2 and V120Slim) have an optional built-in IR filter, and the V100R2 camera has built-in IR illumination.

The focal length of camera lens should be long (i.e., a telephoto lens) because eye images should be captured as large as possible for better recording performance. A 25-mm lens was used for development of the OptiTrack version of SimpleGazeTracker. For development of the Flycapture2 and GPC5300 versions, 10–40mm varifocal zoom lens was used with 1/3-in. and ½-in. sized camera sensors.

Finally, an observer’s head movement has to be restricted using a head rest. Sgttoolbox and SimpleGazeTracker suppose that the relative position of the stimulus presentation monitor, camera unit, and eye are unchanged during recording.

## Installing SimpleGazeTracker and Sgttoolbox

### Single-PC setup

To install SimpleGazeTracker and Sgttoolbox in Single-PC setup, the OS must be Windows or Linux. First install either Matlab or Octave and then install Psychtoolbox-3. Next, download Sgttoolbox from the Sgttoolbox project page (https://sourceforge.net/projects/sgttoolbox/files/). Sgttoolbox consists of two files, SimpleGazeTracker.m and a MEX file. SimpleGazeTracker.m is platform independent, while the MEX file is not. If the platform is Matlab on 64-bit Windows or Octave on 64-bit Linux, download the corresponding MEX file (see Table 2) from the project page. In other environments, the MEX file has to be built from sgttbx_net.c by running the “mex” command on Matlab/Octave. Then, put SimpleGazeTracker.m and the MEX file in the directory that is included in the Matlab/Octave search path. In addition to these files, it is recommended to download a sample file (sample01a.m) to later test installation of Sgttoolbox and SimpleGazeTracker.

**Table 2:** Sgttoolbox files to be downloaded

Platform	Files
Matlab on 64bit Windows	SimpleGazeTracker.m sgttbx_net.mexw64
Octave on 64bit Linux	SimpleGazeTracker.m sgttbx_net.mex
Other	SimpleGazeTracker.m sgttbx_net.c (compile this file to build MEX file)

SimpleGazeTracker can be downloaded from the GazeParser Project page (http://sourceforge.net/projects/gazeparser/files/). The appropriate file depends on the camera used for recording (Table 3). On Windows, double click the file to install. On Linux that supports deb package, use the “dpkg” command to install the package. When using a Linux distribution that does not support deb package, an executable file has to be built from the source package (gzipped tarball). In addition to these files, the camera’s device driver must be installed according to the camera manual. After installation of SimpleGazeTracker and the camera’s device driver, connect the camera to the PC and try to start SimpleGazeTracker. On Windows, SimpleGazeTacker’s icon is created in the Windows Start menu. On Linux, type “sgtsrv” (OpenCV version) or “sgtsrv-flycap” (FlyCapture2 version) from a terminal. Operation of SimpleGazeTracker is described at the GazeParser’s project page.

**Table 3:** SimpleGazeTracker files to be downloaded. x.x.x is SimpleGazeTracker’s version number and y.y.y is the camera library’s version number

OS	Camera library	Files
Windows	OptiTrack	SimpleGazeTracker-x.x.x-OptiTrack-y.y.y.msi
	FlyCapture2	SimpleGazeTracker- x.x.x-FlyCapture2-y.y.y.msi
	GPC5300	SimpleGazeTracker-x.x.x-GPC5300-y.y.y.msi
	OpenCV	SimpleGazeTracker-x.x.x-OpenCV-y.y.y.msi
Linux	FlyCapture2	simplegazetracker-flycap_x.x.x_amd64.deb
	OpenCV	simplegazetracker_x.x.x_amd64.deb

### Dual-PC setup

In Dual-PC setup, one PC is used for stimulus presentation (Presentation PC) and the other is used for recording (Recording PC). The operating system of the Presentation PC can be anything that is supported by PsychToolbox3. On the other hand, the Recording PC must run either Windows or Linux. Both Presentation and Recording PCs must have network adapters and be connected with each other as directly as possible. Using a crossover Ethernet cable is the preferred option, but using a switching hub and normal (i.e., straight) Ethernet cables does not matter in most cases. Setting a fixed IP address for the Recording PC is recommended because Sgttoolbox needs the IP address of the Recording PC when connecting to SimpleGazeTracker. Matlab/Octave and SimpleGazeTracker must not be blocked by a firewall application. The installation procedure for Sgttoolbox and SimpleGazeTracker is the same as that for Single-PC setup, except that Sgttoolbox and SimpleGazeTracker have to be separately installed on the Presentation and Recording PCs.

## Sgttoolbox functions

**Fig. 1:**
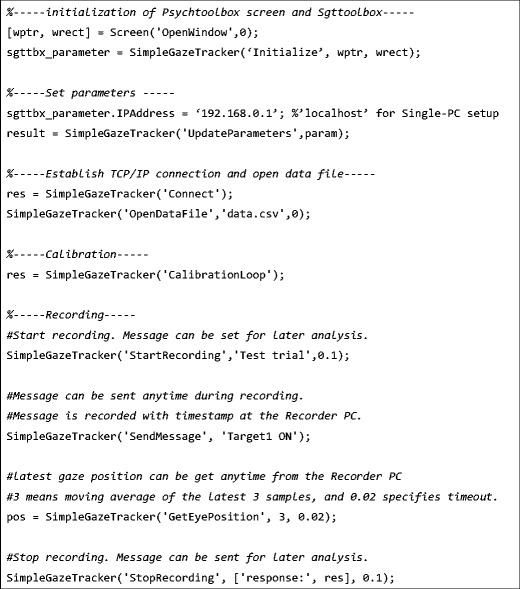
Examples of SimpleGazeTracker() function provided by Sgttoolbox

Sgttoolbox provides only one function named SimpleGazeTracker(). SimpleGazeTracker() is an interface to actual functions of Sgttoolbox, and an actual function can be called by passing a command as the argument of SimpleGazeTracker(). Inserting the SimpleGazeTracker() function into a Psychtoolbox experiment script allows recording of gaze position by SimpleGazeTracker to be synchronized with the experiment. Examples of frequently used commands are shown in Fig. 1. A detailed reference of the commands is available at Sgttoolbox’s project page (http://sgttoolbox.sourceforge.net/reference.html).

In a regular experiment, the “Initialize” command should be called after a Psychtoolbox window is opened. The “Initialize” command returns a structure which holds the Sgttoolbox default parameters. Parameters can be modified by setting new values to the structure and calling the “UpdateParameters” command. After appropriate parameters are set, the “Connect” command can be used to connect Sgttoolbox with SimpleGazeTracker. The “OpenDataFile” command creates SimpleGazeTracker’s data file. The calibration procedure can be started by calling the “CalibrationLoop” command. During the calibration procedure, camera adjustment, calibration, and validating calibration can be performed repeatedly until acceptable accuracy is achieved.

Recording commands are enabled after calibration has been done. The “StartRecording” command makes SimpleGazeTracker write gaze position to the data file. Input from the digital I/O unit is also recorded if the unit is available. During recording, a message string can be embedded in the data file by using the “SendMessage” command. Embedding messages for events (such as stimulus onset and a participant’s key press), these events can be synchronously recorded with gaze data. The latest gaze position data are accessible from the Psychtoolbox script by sending the “GetEyePosition” command. The “StopRecording” command makes SimpleGazeTracker stop writing to the data file. SimpleGazeTracker is running continuously after receiving the “StopRecording” command, and accepts “StartRecording” until SimpleGazeTracker is terminated. By sending “StartRecording” and “StopRecording” during each experimental period (such as during a single trial or a block of trials), data for each period are saved as a separate session in the SimpleGazeTracker data file.

**Fig. 2:**
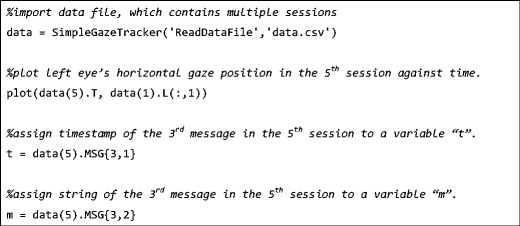
Importing SimpleGazeTracker’s data to Matlab/Octave

In addition to these commands, Sgttoolbox has a command named “ReadDataFile” to import the SimpleGazeTracker’s data file to Matlab/Octave for off-line analysis. This command creates a Matlab/Octave structure from the SimpleGazeTracker’s data file. Table 4 shows members of the structure. If the data file contains multiple sessions, data is imported as a structure array. Figure 2 shows examples of accessing gaze data and embedded messages. Unlike GazeParser, Sgttoolbox does not have a function for detecting saccades. Therefore, users have to write Matlab/Octave codes to detect saccades from the imported data. If users use Python for off-line analysis, they can use GazeParser to analyze a SimpleGazeTracker data file recorded with Sgttoolbox. In such a case, the “SendSettings” command of Sgttoolbox is useful to embed recording conditions (such as the monitor’s physical size, viewing distance and so on) to the SimpleGazeTracker data file.

**Table 4:** Members of Matlab/Octave structure created by “ReadDataFile” command

Member Description	
STARTREC	Date of starting recording (Year, Month, Day, Hour, Minute, Second)
T	Timestamps in milliseconds
L, R	Gaze position of the left and right eye in the screen coordinate. Only single eye data are available if data are recorded in monocular mode
LP, RP	Area of left and right pupil (in number of pixels in the camera image) Available only if SimpleGazeTracker is configured to save pupil area
MSG	Timestamp and string of embedded messages arranged in an Nx2 cell array (N = number of messages). The first and second row correspond timestamp and string, respectively
CAL	Summary of calibration results
PARAM	Parameters embedded in the data file (for compatibility with GazeParser)

## Performance evaluation

In this section, performance tests for Sgttoolbox are described. Because calculation of gaze position from the camera image is carried out solely by SimpleGazeTracker, spatial accuracy and precision of measurement with Sgttoolbox is equal to those values with GazeParser (Sogo, 2013). On the other hand, TCP/IP communication delay for Sgttoolbox would be different from that of GazeParser. Therefore, the communication delays for both Sgttoolbox and SimpleGazeTracker are tested here.

### Method

#### Apparatus

Two PCs referred to as PC1 and PC2 were used for this test. Data recorded by PC1 were used for the performance test. The CPU, motherboard, and graphic board of PC1 was Core i7 2600K (Intel Corp.), DZ68DB (Intel Corp.), and PX9600GT (Leadteck, Inc.), respectively. A 24-in., 60-Hz, Full-HD (1,920×1,080 resolution) monitor was connected for presentation of visual stimuli. Flea3 FL3-U3-13Y3M-C USB3.0 (PointGray, Inc.), OptiTrack V100R2 USB2.0 (NaturalPoint Inc), and IMB-11FT IEEE1394 cameras (imi tech Co., Ltd.) were used for recording. All of these cameras were connected to the motherboard’s interface ports. An onboard LAN port was used for TCP/IP connection in the Dual-PC setup. PC1 was set up in dual-boot configuration of Windows 10 and Ubuntu 14.04LTS. Matlab R2015a was installed in the Windows 10 environment and Octave 3.8.1-1ubuntu1 was installed in the Ubuntu 14.04LTS environment. SimpleGazeTracker 0.9.0 and Sgttoolbox 0.5.0 were installed in both environments.

PC2 was used to test Dual-PC setup only. The CPU and motherboard of PC2 were Core i7 920 (Intel Corp.) and P6T (ASUS Comupter Inc.), respectively. A 24-in., 60-Hz, Full-HD monitor was connected for presentation of visual stimuli. An OptiTrack V100R2 USB2.0 camera was connected to an onboard USB2.0 port. An onboard LAN port was used to connect with PC1. A switching hub and two straight LAN cables were used to connect PC1 and PC2. Windows 10, Matlab R2015a, SimpleGazeTracker 0.9.0, and Sgttoolbox 0.5.0 were installed on PC2.

Table 5 shows all setups used for the tests. FC2, Opti and CV are abbreviations of FlyCapture2 version, OptiTrack version, and OpenCV version, respectively. Dual-Sgttoolbox indicates that PC1 was used for stimulus presentation only. The result of this setup is a baseline of performance of stimulus presentation when SimpleGazeTracker was not running simultaneously on the same PC. FlyCapture2 version was tested on both Windows and Ubuntu to examine effect of OS difference on performance of SimpleGazeTracker. (Note that FlyCapture2 version is an only version that support both of these Oss.) OptiTrack version and OpenCV version were tested as examples of lower-FPS setups comparing with FlyCapture2 version.

**Table 5:** Tested setups

Setups	Role of PC1	OS of PC1	Camera (FPS)
Single-FC2	Presentation/Recording	Win10/Ubuntu	Flea3 (250)
Single-Opti	Presentation/Recording	Win10	V100R2 (100)
Single-CV	Presentation/Recording	Ubuntu	IMB-11FT (60)
Dual-Sgttoolbox	Presentation	Win10/Ubuntu	V100R2 (100)
Dual-FC2	Recording	Win10/Ubuntu	Flea3 (250)
Dual-Opti	Recording	Win10	V100R2 (100)
Dual-CV	Recording	Ubuntu	IMB-11FT (60)

*FC2* FlyCapture2 version, *Opti* OptiTrack version, *CV* OpenCV version

#### Procedure

A person who was experienced in eye-tracking experiments participated in this test. Only a single person was used because the purpose of this test was to evaluate differences between setups. The participant sat on a chair in a dimly-lit room. A head rest was placed in front of the chair to stabilize the participant’s head. The monitor for stimulus presentation was placed at a 57-cm distance from the participant’s eye. The center of the monitor screen was approximately 15 cm below the participant’s eye level. Cameras were placed at approximately a 25 cm distance from the participant’s eyes. Camera angles were adjusted to capture participant’s right eye and not to prevent participant’s sight.

Nine-point calibration was done before the trials were started. At the beginning of a trial, SimpleGazeTracker started recording gaze position. On the stimulus presentation monitor, a white square and a yellow square (0.26×0.26°) were presented on a gray background. The white square moved on the circumference of an imaginary circle of 10.6° diameter placed at the center of the monitor. The starting point of the white square was to the right of the monitor center and moved at 1°/frame of angular velocity in the clockwise direction. If drawing of a visual stimulus did not produce a delay from the monitor’s frame rate (60 Hz), this velocity corresponds to 60°/s of angular velocity. The participant was instructed to track the white square. In every frame, the latest gaze position was obtained by using the “GetEyePosition” command and the yellow square was moved to the gaze position. The run duration of the “GetEyePosition” command and the interval between successive “flip” commands of PsychToolbox was recorded in every frame. A trial was finished after 360 frames were drawn.

To test effects of running Sgttoolbox and SimpleGazeTracker on stimulus drawing performance, the screen was repeatedly filled with gray rectangles of 1,920×1,280 pixels every frame before drawing the white rectangle. Number of repetitions was 1, 4, 16, 64, or 256, and was fixed within a trial. One trial was performed for each of the five drawing loads. This procedure was performed on each of the seven setups shown in Table 5.

## Results and discussion

**Fig. 3:**
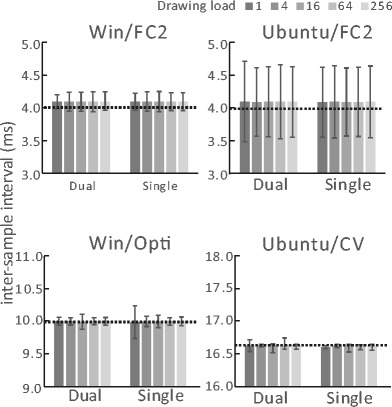
Means and standard deviations of gaze position sampling interval

**Fig. 4:**
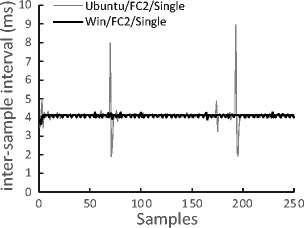
The first 250 samples of raw inter-sample intervals in the Windows and Ubuntu Single-PC setups with FlyCapture2

Figure 3 shows means and standard deviations for gaze position sampling interval recorded by SimpleGazeTracker. Horizontal dotted lines indicate ideal inter-sample intervals calculated from FPS of the camera. Because stimulus drawing and gaze position recording were performed on separate PCs in the Dual-PC setup, it was expected that the sampling interval would not be affected by drawing load. The data shown in Fig. 3 are consistent with this expectation. It is notable that the sampling interval was not affected by the drawing load even in the Single-PC setup. These results indicate that SimpleGazeTracker can record gaze position in the Single-PC setup as well as in the Dual-PC setup. Another noteworthy point is that mean sampling interval of Flycapture2 version (ranging from 4.09 ms to 4.10 ms) was slightly longer than the ideal value (4.0 ms). This indicates that actual sampling rate of the FlyCapture2 version was less than the expected value, as mentioned in “Supported operating systems and camera units.” An interval of 4.10 ms corresponds to about 244 FPS, which may cause problems if a strict 250 FPS sampling rate is necessary. Figure 4 shows the first 250 raw data samples in the Windows and Ubuntu Single-PC setups with FlyCapture2. In Ubuntu, a long interval often occurred and was followed by a short interval. This explains why the standard deviation of the sampling interval was large in Ubuntu FlyCapture2 setups. Because this phenomenon occurs even in the manufacturer’s frame grabber program sample, this would be a performance limitation of the FlyCapture2 library on the current setup.

**Fig. 5:**
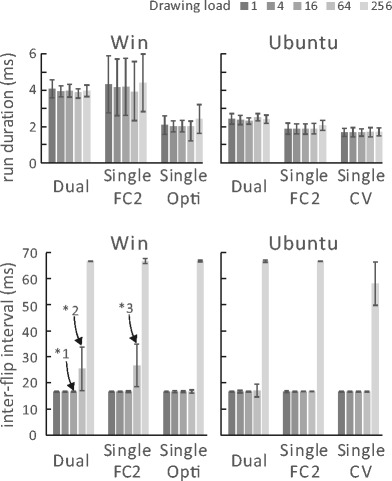
Means and standard deviations of run duration for the “GetEyePosition” command (upper row) and inter-flip interval (lower row). Distributions of inter-flip interval indicated by *1, *2, and *3 are shown in Fig. 6

**Fig. 6:**
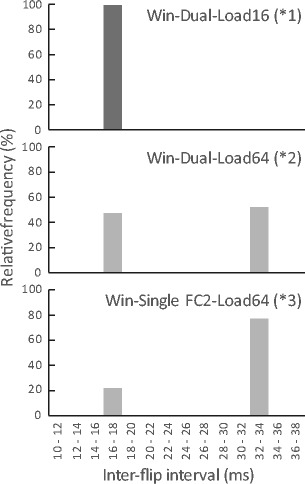
Distribution of inter-flip intervals in the Windows Dual-PC setup at drawing load of 16 (*1), the Windows Dual-PC setup at drawing load of 64 (*2), and the Windows Single-PC setup with FlyCapture2 at drawing load of 64 (*3)

The upper row of Fig. 5 shows the run duration of the “GetEyePosition” command. A shorter run duration is desirable because execution of the script stops during this period. The mean duration reached 4 ms in the Windows Dual-PC setup and Windows Single-PC setup with FlyCapture2. The mean duration was around 2 ms in the other setups. This difference will affect drawing stimulus and detecting participant responses if the processing load is heavy; the bottom row of Fig. 5 depicts this issue. Because the refresh rate of the stimulus presentation monitor was 60 Hz, ideal mean inter-flip interval was 16.7 ms. When drawing load was 256 (256 rectangles were drawn in each frame) mean the inter-flip interval vastly exceeded 16.7 ms in all setups. This was due to a lack of hardware-processing power during this test. When the drawing load was 64, the mean interval exceeded the ideal value only in the setups in which the mean run duration of “GetEyePosition” command reached 4 ms　(i.e., the Windows Dual-PC setup and Windows Single-PC setup with FlyCapture2). To confirm what happened in detail, histograms of inter-flip interval in the setups indicated by *1, *2, and *3 in Fig. 5 are shown in Fig. 6. The top row (*1) of Fig. 6 shows a typical inter-flip interval distribution when the drawing load was less than or equal to 16. The inter-flip interval was narrowly distributed around the ideal value of 16.7 ms. On the other hand, when drawing load was 64 in Windows Dual-PC setup (*2) and Windows Single-PC setup with FlyCapture2 (*3), the inter-flip interval was distributed around both the ideal value and double the ideal value. This indicates that sum of the run duration and drawing time was frequently exceeded in these conditions.

**Fig. 7:**
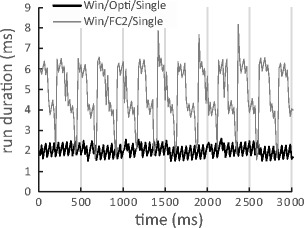
Depiction of 3,000 ms of raw data in Windows Single-PC setup with FlyCpature2 and OptiTrack

There are at least two reasons why the mean run duration was longer in these two setups. Firstly, sending commands to another PC through TCP/IP seemed slow in Windows. On the other hand, the Windows Single-PC setup with the OptiTrack version results suggest that sending commands to the local host was not slow. If this explanation is valid, why was the mean run duration long in the Windows Single-PC setup with FlyCapture2 version? It is possible that the gaze recording load was much heavier in the FlyCapture2 version (250FPS) than in the OptiTrack2 version (100FPS). Figure 7 shows 3,000 ms of raw data from the Windows Single-PC setup, with FlyCapture2 and OptiTrack. Run duration changed periodically in the FlyCapture2 setup, and frequency was slightly higher than 4 Hz (at least 12 cycles in Fig. 7). Possibly this corresponds to the ratio of FlyCapture2’s frame rate (250 FPS) to “GetEyePosition” command frequency (60 Hz). A heavier gaze-recording load would cause “GetEyePosition” command response delay for SimpleGazeTracker in Windows. Both differences in operating systems and gaze recoding loads would cause longer run durations in the Windows Dual-PC setup and Windows Single-PC setup with FlyCapture2.

In conclusion, a PC with Intel 2600K and PX9600GT was capable of running Sgttoolbox and SimpleGazeTracker in the Single-PC setup if stimulus drawing load was not heavy. The graphics board used in this test (PX9600GT) was released in 2008, and newer graphics boards will be able to draw more rectangles in each frame. Sampling frequency of SimpleGazeTracker was stable in both Single-PC and Dual-PC setups.

## Discussion and conclusion

Eye trackers are now more available to researchers, owing to recent releases of low-cost commercial eye trackers. However, considerable effort is still necessary to synchronize eye trackers with stimuli if the library that enables synchronization is not provided. SimpleGazeTracker and Sgttoolbox provide a way to synchronize eye tracking with experiments written by Psychtoolbox, only by adding several lines to the experiment script. They would be a good choice for Psychtoolbox users to start using a gaze-recording experiment.

There are three problems in SimpleGazeTracker and Sgttoolbox. Firstly, they do not have a fixation-detection feature. If fixation detection is not necessary during recording, post-experiment analysis libraries such as EyeMMV toolbox (Krassanakis, Filippakopoulou & Nakos, 2014) can be used to detect fixations. If fixations have to be detected on-line during recording, users have to write codes to retrieve the latest gaze position data from SimpleGazeTracker and analyze it. Although there is no concrete plan to implement an on-line fixation detection feature in Sgttoolbox at present, this would be considered a highly desirable feature to be implemented preferentially.

The second problem is the latency of getting the latest gaze position. As reported above, frame skipping occurred when more than several tens of rectangles were drawn each frame at 60 Hz due to the latency. This severely limits complexity of the visual stimulus drawn in each frame, although using a high performance graphics card relieves this problem.

It is worth noting that the process of getting the latest gaze position consists of three parts, i.e., sending a command by Sgttoolbox, receiving the command, and sending the latest gaze position data by SimpleGazeTracker, and receiving data by Sgttoolbox. On the other hand, Sgttoolbox only has to send the “SendMessage” command when sending event messages such as stimulus onset and participant’s response. This means sending message completes much faster than getting the latest eye position. Therefore, the latency of getting the latest gaze position is problematic mainly when the latest gaze position is necessary every time before a new frame is drawn.

A typical experimental paradigm that needs the latest gaze position at every frame is gaze-contingent stimulus presentation, i.e., changing the stimulus depending on the current gaze position. Because gaze-contingent presentation is an important application of eye tracking systems, it is desirable to reduce the effect of this communication latency. For this purpose, it is planned to add a new feature to Sgttoolbox to receive the latest gaze position in an independent thread from stimulus drawing. This will improve stimulus drawing performance if the CPU has enough processing power.

The third problem is that an observer’s head movement has to be restricted using a head rest. This makes it difficult to use Sgttoolbox and SimpleGazeTracker to record the eye movements of infants, young children, and individuals from many clinical populations. To remove this restriction, some features of SimpleGazeTracker (e.g., supported cameras, high sampling rate) would have to be abandoned. At present, there is no plan to support head-unrestricted recording.

In conclusion, Sgttoolbox is a low-cost option for Psychtoolbox users to record gaze position. In combination with SimpleGazeTracker, gaze recording can be synchronized with stimulus presentation. Events such as target stimulus onset and participant responses can be recorded synchronously with the gaze data.
